# Education Research: Entrustable Professional Activities for General Neurology Advanced Practice Providers: Results of a Modified Delphi Consensus Process

**DOI:** 10.1212/NE9.0000000000200292

**Published:** 2026-01-08

**Authors:** Daniel S. Harrison, Elyse M. Doherty, Cassandra C. Meffert, Christopher T. Doughty, Joel C. Morgenlander, The Entrustable Professional Activities for General Neurology APPs (EPAGNA) Study Group

**Affiliations:** 1 Department of Neurology, Boston University School of Medicine, MA;; 2 Department of Neurology, Brigham and Women's Hospital, Boston, MA;; 3 Department of Neurology, University of Wisconsin, Madison;; 4 Department of Neurology, Mass General Brigham, Boston, MA;; 5 Department of Neurology, Harvard Medical School, Boston, MA; and; 6 Departments of Neurology and Orthopaedic Surgery, Duke University School of Medicine, Durham, NC.

## Abstract

**Background and Objectives:**

A dedicated didactic framework, assessment strategy, and consensus expectations for advanced practice providers (APPs) entering general neurology practice for the first time have not been described. We aimed to define entrustable professional activities (EPAs) for general neurology APPs and to provide further validity evidence for the EPAs through application of the EQual rubric.

**Methods:**

This was a modified Delphi consensus process. Panelists were leaders of neurology APP fellowship programs and other established experts in neurology APP education. The steering committee identified putative EPA topics. Panelists voted on a 5-point Likert scale how important it was that a new general neurology APP be able to perform specific activities with indirect supervision remotely available by the end of their on-the-job training. Panelists were allowed to propose modifications to putative EPAs and suggest new EPAs. After 3 rounds of voting, full EPA descriptions were drafted by the steering committee. Full EPA descriptions were sent to external experts in neurology APP education for assessment of their structure and quality. The steering committee met again to discuss feedback from the external experts and make adjustments as needed. The full EPA descriptions were sent to the Delphi panelists for a final round of voting.

**Results:**

Of 35 experts invited to participate in the Delphi process, 30 agreed to serve as panelists, 16 of whom were program leaders in neurology APP fellowship programs. The steering committee proposed 13 core and 52 nested EPA topics and the panelists proposed 6 modifications and an additional 4 nested EPAs. After 3 rounds of voting, 13 core and 46 nested EPAs were retained and full EPA descriptions were authored. All EPA descriptions met the pre-specified cut score for quality and structure and were retained in a final Delphi round. Overall entrustment expectations did not differ between panelists who were fellowship program leaders and those who were not (5-point Likert median [interquartile range], 4 [4–5] vs 4 [4–5], *p* = 0.980, *r* = 0.005).

**Discussion:**

These consensus EPAs may be applied for curricular development and assessment for new general neurology APPs. Entrustment expectations did not differ between those who were leaders in fellowship programs and those who were not.

## Introduction

The wait time to see a neurologist has increased over the last decade, suggesting that the shortage of neurologists in the United States is getting worse as projected.^1-3^ In the same time frame, the number of physicians self-identifying as general neurologists has dropped from 46% to 28%, suggesting that general neurology may be especially burdened by the shortage.^4^ Incorporation of advanced practice providers (APPs) in neurology has been proposed as a key strategy to mitigate this shortage.^5-7^ Indeed, APP practice has grown and is projected to expand further in the next decade.^8,9^ However, most graduates of APP degree programs have limited exposure to clinical neurology training.^10,11^ This means that new neurology APPs need additional clinical training, whether through on-the-job training or fellowship programs, after completing their degree programs. What this postgraduate training should entail, assessment strategies during this period, and expectations of new general neurology APPs upon completion of this training, however, have not been described.

Milestones are in place as a developmental and assessment framework for physician training in general neurology.^12^ However, the timeframe and goals of general neurology resident and APP training are distinct, limiting direct application of the milestones for new general neurology APPs. The entrustable professional activities (EPAs) framework, which has been proposed as a useful concept in neurology education, may be more directly applicable to APP practice.^13^ EPAs are units of professional practice to be entrusted to an individual once they have attained sufficient, specific competence. Although milestones are used exclusively in physician graduate medical education, EPAs have been developed and implemented for other groups of new postgraduate APPs.^14,15^ Development of a didactic framework for new general neurology APPs would facilitate training and assessment for this group of clinicians. As such, we aimed to define EPAs for general neurology APPs using a modified Delphi consensus process and to provide further validity evidence for the EPAs through application of the EQual rubric.^16^

## Methods

This was a modified Delphi consensus process with 3 prespecified rounds (Figure).^17^ The steering committee (the author group) included physicians and APPs who are leaders in APP fellowship and on-the-job training programs with previous experience in neurology APP education research, including Delphi methodology. The steering committee performed a literature review to identify putative EPA topics from EPAs, observable practice activities, and milestones from other learner groups.^12,14,18-20^ A single member of the steering committee (D.S.H.) identified the relevant literature, which was then reviewed by all members of the steering committee. The steering committee subsequently developed the list of putative EPA topics iteratively. These were then sorted into “core” and “nested” EPAs and transformed into a Delphi questionnaire.^21^ All Delphi rounds took place online and anonymously through Qualtrics over approximately 10 days. Delphi panelists were recruited from 2 groups—leaders of neurology APP fellowship programs and other established experts in neurology APP education. Other experts were published authors in neurology APP education or practice, leaders of a national/international committee on neurology APP education or practice, invited speakers on APP education or practice for a national/international audience, and institutional APP program leaders. No more than 2 panelists from a single institution were invited to participate. Panelists voted on a 5-point Likert scale (from “not important” to “mandatory”) on how important it was that a new general neurology APP be able to perform specific activities with indirect supervision remotely available by the end of their on-the-job training. For APPs who had completed a neurology fellowship program, this included on-the-job training after completion of the fellowship. This level of supervision (level 4 of a generic entrustment-supervision scale) was selected for voting by consensus of the steering committee as a pragmatic goal of on-the-job training for new neurology APPs.^22-24^ Although level 3 (permission to perform an activity with indirect supervision immediately available) may be the goal of on-the-job training in select contexts, there were clear cases in which this would be inappropriate (e.g., in the training of a new nocturnist APP).

**Figure:**
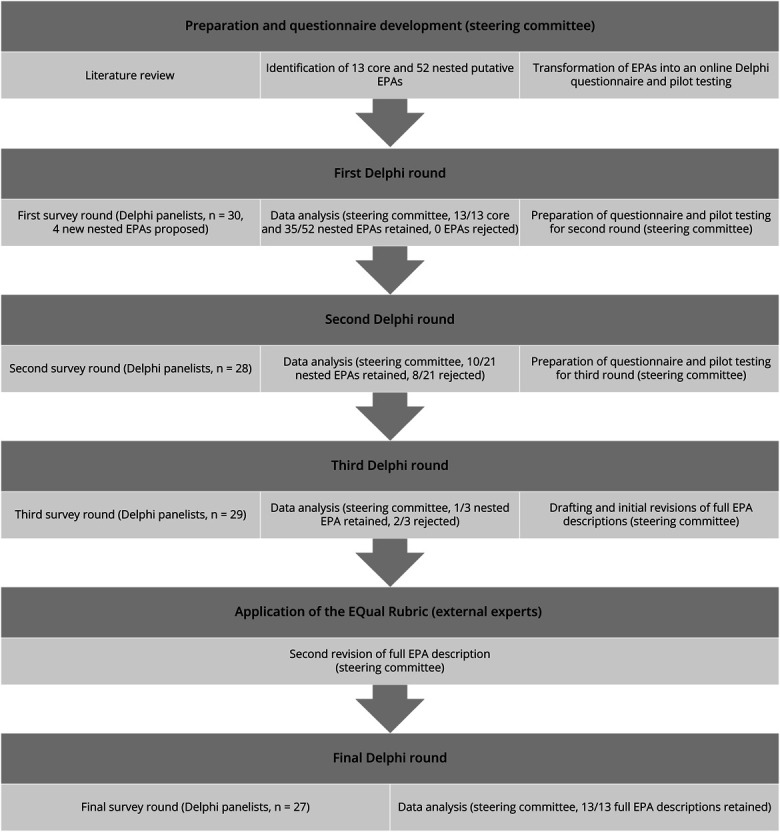
Study Flowchart EPA = entrustable professional activity.

During the first round of voting, panelists were allowed to propose modifications to putative EPAs and suggest new EPAs. After each round, the steering committee analyzed the panelists' responses and prepared the Delphi questionnaire for the next round. All panelists were provided with their votes from the previous round and the average and SD of the panel's vote on each item. Items undergoing a first round of voting which received ≥80% “very important” or “mandatory” votes were retained. Items with ≤20% “very important” or “mandatory” votes were rejected. All other items were voted on in a second round. Items undergoing a second round of voting were retained if they received ≥80% “very important” or “mandatory” votes. All other items were rejected. Items which were modified based on suggestions from the panel were treated as new items in the subsequent round of voting.

After 3 rounds of voting, full EPA descriptions were drafted by the steering committee.^25^ The full EPA descriptions were sent to 2 external experts in neurology APP education for assessment using the EQual rubric.^16^ The EQual rubric is a highly reliable, 14-item instrument for assessing the quality and structure of EPAs. Each item is scored on a 5-point scale. After application of the EQual rubric, the steering committee met again to discuss feedback from the external experts and make adjustments as needed. Finally, the full EPA descriptions were sent to the Delphi panelists for a final round of voting with the same rules for item retention or rejection as in the first Delphi round.

### Statistical Analysis

Delphi round voting was analyzed descriptively. Average item ratings from the EQual rubric were compared against an a priori cut score of 4.07 as previously described.^16^ SD between rounds was compared using a paired *t* test with Cohen *d* as a measure of effect size. An exploratory analysis was conducted to compare entrustment expectations of panelists who were APP fellowship program leaders to those not affiliated with fellowship programs using Mann-Whitney U tests with Bonferroni correction and effect size calculated as described by Rosenthal.^26^ All statistical analyses were conducted with SPSS version 29.0.2.0.

### Standard Protocol Approvals, Registrations, and Patient Consents

This study was approved by the Boston University institutional review board. Consent to participate in the study was implied by completion of the questionnaires.

### Data Availability

Anonymized data not published within this article will be made available by request from any qualified investigator.

## Results

Of 35 experts invited to participate in the Delphi process, 30 agreed to serve as panelists, 16 of whom were program leaders in neurology APP fellowship programs. Of the panelists, 13 were physicians, 9 were nurse practitioners, 7 were physician assistants, and 1 was a clinical nurse specialist. Panelists represented all 4 regions and 8 of 9 divisions of the United States. Nineteen panelists (63.3%) were women. Qualifications of the Delphi panelists are summarized in Table 1. All 30 panelists participated in the first round. There was some panelist attrition, with between 27 and 29 panelists participating in the following rounds (Figure).

**Table 1:** Delphi Panelist Characteristics and Qualifications

Characteristic/qualification	Average ±SD or n (%)
Panelist training	
Physician	13 (43.3)
Nurse practitioner	9 (30.0)
Physician assistant	7 (23.3)
Clinical nurse specialist	1 (3.3)
Practice setting^a^	
Outpatient	24 (80.0)
Inpatient	17 (56.7)
Critical care	6 (20.0)
Years in practice	17.6 ± 9.2
Neurology APPs taught and trained	14.4 ± 10.2
Fellowship program director	9 (30.0)
Fellowship associate/assistant director	5 (16.7)
Medical director for department or division APP program	4 (13.3)
Senior, lead, or chief neurology APP	9 (30)
Invited national speaker on neurology APP practice or education	14 (46.7)
National committee service for neurology APP practice or education	11 (36.7)
Role in hiring new neurology APPs	23 (76.7)

Abbreviation: APP = advanced practice provider.

a Participants were instructed to select all that applied.

The steering committee proposed 13 core and 52 nested EPA topics (eAppendix 1). After 1 round of voting, 13 core and 35 nested EPAs were retained, no putative EPAs were rejected, and 17 nested EPAs required an additional round of voting. Modifications were applied to 6 of these items based on panelist suggestions, and the panel proposed an additional 4 nested EPAs. After the second round of voting, 10 nested EPAs were retained, 8 nested EPAs were rejected, and 3 nested EPAs required an additional round of voting. After the third round of voting, 1 nested EPA was retained and 2 were rejected. Of items which underwent an additional round of voting, average SD of panelist responses decreased (mean [SD], 0.80 [0.08] vs 0.69 [0.08], *p* < 0.001, *d* = 1.37), supporting evolution of consensus.

Upon application of the EQual rubric, each full EPA description met the pre-specified cut score for quality and structure (Table 2). In the final round of voting, all 13 full EPAs descriptions were retained (eAppendix 2). Of the original 65 steering committee proposed core and nested EPAs, 46 were retained without modification.

**Table 2:** EQual Scores for Full Entrustable Professional Activity Descriptions

Entrustable professional activity title	EQual score
1. Communicating and collaborating as a member of an interprofessional team to care for a patient with a neurologic problem	4.21
2. Counseling patients and their families about neurologic diseases	4.50
3. Documenting a clinical neurologic encounter in the patient record	4.68
4. Forming clinical neurology questions and retrieving evidence	4.18
5. Gathering a neurologic history	4.79
6. Giving and receiving handover of a patient with a neurologic problem to transition care	4.75
7. Localizing a lesion	4.32
8. Performing a neurologic exam	4.82
9. Prioritizing a differential diagnosis for a patient with a neurologic problem	4.46
10. Providing an oral presentation of a neurologic encounter	4.50
11. Recognizing a patient requiring urgent or emergent neurologic care and initiating evaluation and management	4.39
12. Recommending and interpreting tests for a patient with a neurologic problem	4.54
13. Recommending treatments for a patient with a neurologic problem	4.46

Overall entrustment expectations did not differ between panelists who were fellowship program leaders and those who were not (5 point Likert median [interquartile range], 4 [4–5] vs 4 [4–5], *p* = 0.980, *r* = 0.005). After correcting for multiple comparisons, entrustment expectations for the individual core and nested EPAs did not differ between groups (eAppendix 1).

## Discussion

We describe consensus EPAs for new general neurology APPs, provide evidence supporting their quality and structure, and demonstrate that entrustment expectations do not differ between clinicians who are neurology APP fellowship program leaders and those who are not. These EPAs may be applied immediately for curricular development and assessment for new general neurology APPs.

Regarding curricular development, these EPAs serve as a guideline for what a new general neurology APP should be able to do with indirect supervision remotely available after completion of on-the-job training. For some of these EPAs, such as “communicating and collaborating as a member of an interprofessional team to care for a patient with a neurologic problem,” new neurology APPs may obtain sufficient, specific competence through clinical experience. For others, learning activities supplementing clinical experience are likely required. For example, a new nocturnist general neurology APP practicing at a center which admits on average 2 patients per year with Guillain-Barre syndrome (GBS) may not care for a patient with this diagnosis over the course of their on-the-job training program. However, it would clearly be beneficial for this individual to have had specific training in recognizing and initiating treatment of a patient with GBS (as in EPAs 9, 11, and 13) before performing these activities with only indirect supervision remotely available. In this way, the EPAs can guide which learning activities should be included to supplement clinical experience during on-the-job training to prepare a new general neurology APP for practice with this level of supervision. In the context of neurology APP fellowship training, as the number of programs grow, this didactic framework will promote consistency between programs, which will be important for applicants, trainees, potential employers, and most of all patients.

EPA-based assessments are simple to construct and correlate with performance, including in the neurology APP population.^23,27,28^ This is in contrast with milestone-based assessments, which do not consistently correlate with performance.^29^ Implementation of this EPA framework for assessment of new general neurology APPs would facilitate competency-based (as opposed to time-based) transitions to more independent practice. Postgraduate training for neurology APPs is not bound by the same historically rigid, time-based promotion structure of physician training. Application of these EPAs for assessment could be advantageous for patient safety (in ensuring APPs who are promoted to decreasing levels of supervision have demonstrated sufficient, specific competence) and for health care cost reduction (in ensuring that APPs who are ready for more independence are not “held back” in on-the-job training longer than necessary). Although assessments based on these EPAs may be simple to construct, the EPAs themselves are not assessment tools. Future work should aim to support EPA-based assessments for general neurology APPs with validity evidence, especially when the intent of assessment is summative. In addition, these EPAs could serve as a blueprint for a standardized test which could be offered to fellowship program graduates as well as experienced neurology APPs to obtain credentials as a certified neurology APP. Although not every neurology APP should be required to obtain such a certification, it would signal to potential employers that an applicant has demonstrated minimum competency based on consensus standards. Diplomates themselves might gain a competitive advantage in the job market and in contract negotiations.

Entrustment expectations did not differ between those who were leaders in fellowship programs and those who were not. Although one might expect that program leaders would have had overall higher expectations of their graduates given the addition of fellowship training to standard on-the-job training, this finding reflects that, regardless of training pathway, the same knowledge, skills, and attitudes are required to care for a common patient population. Although entrustment expectations did not differ, this does not necessarily imply that these 2 training pathways are equally successful in producing competent general neurology APPs. Whether 1 pathway is more effective in fostering competence or is more cost-effective should be the subject of future investigation.

There were several notable omissions from the final list of EPAs. Lumbar puncture, botulinum toxin injection, and other common procedures in neurologic practice were not included. This is not to say that the steering committee or expert panel believed that APPs should not be entrusted to perform procedures (in our experience, APPs are sometimes the most competent procedural operators and teachers). An item for “performing procedures” was not proposed by the steering committee because the specific procedures entrusted to an APP likely vary greatly by practice setting and because a typical onboarding program may not be of sufficient duration to achieve the pre-specified level of entrustment. Other items may similarly have been rejected because they require more practice than a typical new APP is exposed to during an onboarding program (e.g., performing a fundoscopic examination and identifying papilledema) or because they are typically delegated to subspecialty neurologists and APPs (e.g., recommending treatments for a patient with a brain tumor).

Limitations of these EPAs include that they were developed for general neurology APPs and cannot be directly applied for training of subspecialty neurology APPs. An APP practicing in a headache clinic, for example, may be required to perform procedures that are not typically required of general neurology APPs are thus not reflected in these EPAs. Educators from neurologic subspecialties in which APP practice is common should consider developing unique EPAs for their new APPs.^14^ On the other hand, the learner group to which these EPAs should be applied may be interpreted as too heterogeneous. As above, the EPAs were designed to be applied to both fellowship training and traditional on-the-job training because the same knowledge, skills, and attitudes are required to care for a common patient population. There may be some activities (for example, “performing a lumbar puncture”) which would have been included if the EPAs were only meant to apply to inpatient general neurology APPs and vice versa. Ultimately, the steering committee determined that unified consensus inpatient and outpatient general neurology EPAs were most generalizable. Virtually all panelists were from academic medical centers, potentially limiting the generalizability of these findings to community and private practice. It is possible that entrustment expectations of neurology APPs in smaller practice settings with fewer resources are higher than those identified by the panel due to relative lack of access to subspecialty neurologists. However, agreement between panelists from academic and community practice was high in a prior study of EPA development for neurology APPs.^14^ The full list of core and nested EPAs is relatively long, which may limit its applicability for assessment. Finally, there was a small amount of panelist attrition between Delphi rounds. It is possible that if every panelist voted in every round, some retained items would have been rejected and vice versa.

These consensus EPAs may be applied for curricular development and assessment for new general neurology APPs. Entrustment expectations did not differ between those who were leaders in fellowship programs and those who were not.

## Appendix Coinvestigators

**Table:**

Name	Location	Role	Contribution
Shannon Anderson, MPAS, PA-C	Oregon Health and Science University, Portland	Expert panelist	Provided expert opinion on putative EPAs, reviewed manuscript
Jennifer Cheng, NP	Massachusetts General Hospital, Boston	Expert panelist	Provided expert opinion on putative EPAs, reviewed manuscript
Calli L. Cook, DNP, APRN, FNP-C, FAANP, FAAN	Nell Hodgson Woodruff School of Nursing, Emory University, Emory Brain Health Center, Atlanta	Expert panelist	Provided expert opinion on putative EPAs, reviewed manuscript
Salvador Cruz-Flores, MD	Texas Tech University Health Science Center, El Paso	Expert panelist	Provided expert opinion on putative EPAs, reviewed manuscript
John S. Emmett, PA-C	Indiana University Health, Indianapolis	Expert panelist	Provided expert opinion on putative EPAs, reviewed manuscript
John Engstrom, MD	University of California San Francisco	Expert panelist	Provided expert opinion on putative EPAs, reviewed manuscript
Miriam L. Freimer, MD	The Ohio State University Wexner Medical Center, Columbus	Expert panelist	Provided expert opinion on putative EPAs, reviewed manuscript
Scott M. Friedenberg, MD	Geisinger, Danville	Expert panelist	Provided expert opinion on putative EPAs, reviewed manuscript
Gwen A. Garden, MD, PhD	University of North Carolina at Chapel Hill, Chapel Hill	Expert panelist	Provided expert opinion on putative EPAs, reviewed manuscript
Julia Gray, MSN, CRNP	University of Alabama at Birmingham	Expert panelist	Provided expert opinion on putative EPAs, reviewed manuscript
Julie A. Gurwell, PhD, PA-C	University of Kentucky, Lexington	Expert panelist	Provided expert opinion on putative EPAs, reviewed manuscript
Allyson Hamacher, PA-C	Mayo Clinic Arizona, Scottsdale	Expert panelist	Provided expert opinion on putative EPAs, reviewed manuscript
Jason Johns, MPAS, PA-C	Dartmouth Hitchcock Medical Center, Lebanon	Expert panelist	Provided expert opinion on putative EPAs, reviewed manuscript
Keri Johnson, RN, MSN, FNP	University of Virginia Health, Charlottesville	Expert panelist	Provided expert opinion on putative EPAs, reviewed manuscript
Chaouki Khoury, MD	PANDA Neurology, Atlanta Headache Specialists, Atlanta	Expert panelist	Provided expert opinion on putative EPAs, reviewed manuscript
Alexander W. King, AG-PCNP	University of Virginia Health, Charlottesville	Expert panelist	Provided expert opinion on putative EPAs, reviewed manuscript
Pearce Korb, MD, MHPE, FAAN	Virginia Commonwealth University School of Medicine, Richmond	Expert panelist	Provided expert opinion on putative EPAs, reviewed manuscript
Ashley Lengel, MS, PA-C	Duke University School of Medicine, Durham	Expert panelist	Provided expert opinion on putative EPAs, reviewed manuscript
Terri Milburn, NP-C	Emory University, Atlanta	Expert panelist	Provided expert opinion on putative EPAs, reviewed manuscript
Jamelah A. Morton, APRN	Miami Neuroscience Institute, Baptist Health South Florida, Miami	Expert panelist	Provided expert opinion on putative EPAs, reviewed manuscript
Darcy O'Banion, CNS, MS	University of Colorado School of Medicine, Aurora	Expert panelist	Provided expert opinion on putative EPAs, reviewed manuscript
Victoria Pelak	University of Colorado School of Medicine, Aurora	Expert panelist	Provided expert opinion on putative EPAs, reviewed manuscript
Amy Perrin Ross, APN, MSN, MSCN, CNRN	Loyola University Medical Center, Chicago	Expert panelist	Provided expert opinion on putative EPAs, reviewed manuscript
Aashit K. Shah, MD	Virginia Tech Carilion School of Medicine, Roanoke	Expert panelist	Provided expert opinion on putative EPAs, reviewed manuscript
Gretchen E. Tietjen, MD	University of Toledo College of Medicine and Life Sciences	Expert panelist	Provided expert opinion on putative EPAs, reviewed manuscript
Karen Tillotson, DHSc, PA-C	Geisinger, Wilkes Barre	Expert panelist	Provided expert opinion on putative EPAs, reviewed manuscript
Andrea Thurler, DNP, FNP-BC	Massachusetts General Hospital, Boston	Expert panelist	Provided expert opinion on putative EPAs, reviewed manuscript
Diego R. Torres-Russotto, MD, FAAN	Miami Neuroscience Institute, Baptist Health South Florida	Expert panelist	Provided expert opinion on putative EPAs, reviewed manuscript
Juliana H. VanderPluym, MD, FRCPC, FAHS	Mayo Clinic Arizona, Scottsdale	Expert panelist	Provided expert opinion on putative EPAs, reviewed manuscript
Brannon Vines, MD	University of Alabama at Birmingham	Expert panelist	Provided expert opinion on putative EPAs, reviewed manuscript

## Glossary

APP: advanced practice provider
EPA: entrustable professional activity
GBS: Guillain-Barre syndrome
